# Long-lasting alterations to DNA methylation and ncRNAs could underlie the effects of fetal alcohol exposure in mice

**DOI:** 10.1242/dmm.010975

**Published:** 2013-04-10

**Authors:** Benjamin I. Laufer, Katarzyna Mantha, Morgan L. Kleiber, Eric J. Diehl, Sean M. F. Addison, Shiva M. Singh

**Affiliations:** 1 Molecular Genetics Unit, Department of Biology, Western University, London, Ontario, N6A 5B7, Canada

## Abstract

Fetal alcohol spectrum disorders (FASDs) are characterized by life-long changes in gene expression, neurodevelopment and behavior. What mechanisms initiate and maintain these changes are not known, but current research suggests a role for alcohol-induced epigenetic changes. In this study we assessed alterations to adult mouse brain tissue by assaying DNA cytosine methylation and small noncoding RNA (ncRNA) expression, specifically the microRNA (miRNA) and small nucleolar RNA (snoRNA) subtypes. We found long-lasting alterations in DNA methylation as a result of fetal alcohol exposure, specifically in the imprinted regions of the genome harboring ncRNAs and sequences interacting with regulatory proteins. A large number of major nodes from the identified networks, such as *Pten* signaling, contained transcriptional repressor CTCF-binding sites in their promoters, illustrating the functional consequences of alcohol-induced changes to DNA methylation. Next, we assessed ncRNA expression using two independent array platforms and quantitative PCR. The results identified 34 genes that are targeted by the deregulated miRNAs. Of these, four (*Pten*, *Nmnat1*, *Slitrk2* and *Otx2)* were viewed as being crucial in the context of FASDs given their roles in the brain. Furthermore, ∼20% of the altered ncRNAs mapped to three imprinted regions (*Snrpn-Ube3a*, *Dlk1-Dio3* and *Sfmbt2*) that showed differential methylation and have been previously implicated in neurodevelopmental disorders. The findings of this study help to expand on the mechanisms behind the long-lasting changes in the brain transcriptome of FASD individuals. The observed changes could contribute to the initiation and maintenance of the long-lasting effect of alcohol.

## INTRODUCTION

An epigenetic mechanism brings about changes in gene expression or cellular phenotype through changes other than a change in the underlying DNA sequence. They are crucial to gene regulation at two distinct levels. The first is at the level of transcription, which is mainly accomplished by methylation of DNA (Métivier et al., 2008) and modification of histones. The second level of epigenetic control is achieved post-transcriptionally by small noncoding RNAs [ncRNAs; i.e. microRNAs (miRNAs)] and allows for the fine-tuning of gene expression (Moazed, 2009). miRNAs are key regulators of eukaryotic gene expression, acting via translational repression and mRNA decay (Friedman and Jones, 2009). Furthermore, miRNAs control the activity of more than 60% of all protein-coding genes in mammals and are involved in the regulation of most cellular processes (Fabian et al., 2010).

The two aforementioned levels of epigenetic regulation are particularly crucial during embryonic development (Howlett and Reik, 1991; Monk et al., 1987), where changes to the epigenome are tightly controlled (Howlett and Reik, 1991; Monk et al., 1987). Alcohol consumption during pregnancy results in the development of fetal alcohol spectrum disorders (FASDs) (May and Gossage, 2001). Phenotypes commonly associated with FASD include impairments in cognition, learning, executive function, judgment, attention and social adaptation (Herman et al., 2008; Jirikowic et al., 2008; Mattson et al., 1998). FASDs are relatively common in North America, affecting 2–5% of pregnancies, and have an annual cost in the tens of billions of dollars (Chudley et al., 2005; Lupton et al., 2004; May et al., 2009; Popova et al., 2011; Stade et al., 2009).

Insight into the effect of alcohol on the developing brain (Valenzuela et al., 2012) has been assessed using animal models, including rats for neuroscience based studies and the C57BL/6J (B6) strain of mice for molecular-biology-based studies. Previously, we reported that a mouse model for FASD based on voluntary maternal alcohol consumption throughout gestation resulted in offspring that showed mild developmental delay, anxiety-related traits and deficits in spatial learning (Kleiber et al., 2011). Next, we extended this model by evaluating the gene expression changes that occurred in the adult brains of C57BL/6J mice that were prenatally exposed to alcohol via maternal preference drinking (Kleiber et al., 2012). The results indicated that alcohol induces subtle but consistent changes to global gene expression. Gene enrichment analysis showed over-represented gene ontology classifications of cellular, embryonic and nervous system development. Furthermore, a number of genes identified have previously been implicated in FASD-relevant neurobehavioral phenotypes such as cognitive function, anxiety, attention deficit hyperactivity disorder and mood disorders (e.g. *Otx2*).

In our most recent experiments we have sought to examine how the variability seen in FASD phenotypes relates to the timing of alcohol exposure. In these experiments, mice were exposed to two acute doses of alcohol (5 g/kg body weight) at neurodevelopmental times representing the human first, second or third trimester equivalent. This method has been previously reported and induces a peak blood alcohol level of over 0.3 g/dl for 4–5 hours following injection, and is sufficient to induce neuronal apoptosis and result in FASD-related behaviors (Ikonomidou et al., 2000; Wozniak et al., 2004).

**TRANSLATIONAL IMPACT**

**Clinical issue**

Birth defects caused by maternal consumption of alcohol during pregnancy are collectively termed fetal alcohol spectrum disorders (FASDs). Although the disorders are entirely preventable, they remain the leading cause of cognitive deficits in North America, highlighting the need to elucidate the underlying mechanisms and develop effective treatments. Studies using animal models of FASD have shed light on the effects of alcohol on the developing brain, and alcohol-induced changes in global gene expression have been reported. It has been proposed that long-term changes in the expression of specific gene modules, mediated by epigenetic mechanisms, are a key feature of FASDs, but the specific processes involved have not yet been determined.

**Results**

The authors of this report previously generated and characterized mouse models of FASD and observed alterations in post-natal development, adult behavior and gene expression. In this study, they sought to determine the mechanisms underlying the long-term alterations in brain gene expression following alcohol exposure during development. To this end, they examined the effects of fetal alcohol exposure on the epigenome of adult mice by assaying DNA methylation and non-coding RNA (ncRNA) expression. They observed alterations in DNA methylation and, by applying bioinformatics tools, identified the specific genomic regions affected. The changes mapped to promoters of small ncRNA molecules that are normally regulated in a parent-of-origin manner via epigenetic mechanisms. These molecules are known regulators of many genes, some of which have been previously implicated in FASD. Using array platforms and quantitative PCR, the group pinpointed 34 genes that are targeted by the deregulated ncRNA molecules, providing a suite of candidates for further analysis. Importantly, the authors demonstrate that the alterations in DNA methylation mediated by fetal alcohol exposure are long lasting.

**Implications and future directions**

The results of this study provoke a number of novel hypotheses and candidate molecular markers for FASD. Specifically, the study has identified key genomic regions and regulatory relationships that warrant further research and validation to uncover currently unknown functions driving the persistence of FASD into adulthood. Interestingly, the small RNA molecules implicated are known to be largely brain-specific and have been linked with a number of complex neurological disorders. Overall, the results suggest that a multistage process involving changes in DNA methylation, ncRNAs and gene expression underlies FASD and related diseases of fetal origin. Given the highly reversible nature of epigenetic markers, the newly identified markers from this work have great potential for use in FASD treatment.

In our first set of experiments we set out to examine the physiological, developmental and behavioral deficits associated with the paradigms examined (Mantha et al., 2013). The results showed that alcohol exposure at any time during gestation causes delays in motor skill and reflex development. Alcohol exposure during the trimester three equivalent seems to produce delays in most measures, followed closely by trimester two, whereas alcohol exposure at the first trimester producing subtle effects, with less than half of the milestones significantly altered. Finally, our results provided further support for deficits in learning and memory caused by prenatal alcohol exposure, as assessed by the Barnes maze.

After the battery of developmental milestones, physiological metrics and behavior work, the mice were reared to adulthood and changes to their adult brain transcriptome were assessed in a second group of experiments (Kleiber et al., 2013). The results suggested that alcohol disrupts biological processes that are actively occurring at the time of exposure. These include cell proliferation during trimester one, cell migration and differentiation during trimester two, and cellular communication and neurotransmission during trimester three. Furthermore, although alcohol altered a distinct set of genes depending on developmental timing, many of these show interrelatedness and can be associated with one another via ‘hub’ molecules and pathways.

Ultimately, the findings of our most recent experiments argue that long-term changes in the expression of specific gene modules represent the major feature of the effect of alcohol during the development of FASD. Our third set of experiments, which form the basis of this manuscript, seek to examine the mechanisms initiating and maintaining the long-term gene expression changes following fetal alcohol exposure (FAE). Currently, these mechanisms are unknown; however, recent studies by others have suggested that epigenetic alterations could underlie the effects seen in our first two sets of experiments (Govorko et al., 2012; Haycock and Ramsay, 2009; Kaminen-Ahola et al., 2010; Liu et al., 2009). Indeed, alcohol exposure during embryonic development affects the transfer of folate from the mother to the developing embryo (Hutson et al., 2012). This is of significance to the developing embryo because folate is essential in establishing and maintaining DNA methylation. The lack of folate has the potential to cause aberrant epigenetic profiles. Indeed, recent studies have observed methylation changes occurring in genes that are known to be genomically imprinted (Dietz et al., 2012; Liu et al., 2009; Shukla et al., 2011; Sittig et al., 2011). Imprinted genes are expressed in a parent-of-origin-specific manner that is based on differential methylation of an imprinting control region (ICR). Genomic imprinting acts only on a select set of genes (Laprise, 2009; Moore, 2001) that are important in early development, particularly neurodevelopment (Hager and Johnstone, 2003). These genes are crucial during neurodevelopment, as well as in the normal functioning of the brain (Davies et al., 2008). Interestingly, ∼30% of imprinted genes are hypothesized to be ncRNAs, including miRNAs (Morison et al., 2005). miRNAs have been implicated in a number of abnormalities that often show co-morbidity with FASD, including anxiety, depression and other psychiatric disorders (O’Connor et al., 2012). Approximately 11% (42/385) of examined miRNAs have been found to be differentially expressed in primary cortical neuronal cultures chronically exposed to alcohol (Guo et al., 2011). Interestingly, treatments that contain methyl group donors, such as choline (Zeisel, 2011), have been able to attenuate some of the effects of FAE. Of particular interest to this research is the observation that co-incubation of alcohol-exposed mouse embryos with folic acid, which is involved in establishing DNA methylation, was able to prevent altered expression of *mir-10a* and its target gene *Hoxa1* (Wang et al., 2009). Such results argue that the alcohol-induced molecular cascade (Soares et al., 2012; Wang et al., 2009) might involve DNA methylation. However, the actual mechanism behind such interactions remains to be elucidated (Haycock and Ramsay, 2009; Miranda, 2012).

It is logical to hypothesize that FAE might alter DNA methylation, thus suppressing the transcriptional machinery involving a variety of transcription factors. For example, CTCF is a highly conserved ubiquitous 11-zinc-finger protein with multiple functions in chromatin organization and gene regulation, including chromatin insulator activity and transcriptional enhancement and silencing (Williams and Flavell, 2008). CTCF binds to a set of signal sequences, with this binding being sensitive to methylation (Filippova, 2008), while also having the potential to mediate long-range chromosomal interactions (Ling et al., 2006). Currently, 111,062 CTCF-binding sites in the mouse genome have been identified, with some being tissue specific and some ubiquitous (Shen et al., 2012). Indeed, CTCF-binding sites involved in the ICR of *H19* and *Igf2* (*H19/Igf2*) have shown significant differential methylation in FASD placental tissue (Haycock and Ramsay, 2009) and in the sperm of alcohol-consuming fathers (Knezovich and Ramsay, 2012). Furthermore, altered DNA methylation has the potential to directly or indirectly affect the expression of a specific set of miRNAs that could play a substantial role in maintaining long-term alterations in the gene expression.

The results presented in this report include three novel findings. First is the disruption of genome-wide DNA methylation in adult brain tissue. Second are the associated alterations in the expression of miRNAs and small nucleolar RNAs (snoRNAs). Third, we present the use of bioinformatic tools to identify specific (imprinted) genomic regions and their interactive protein, CTCF, which might initiate and maintain abnormalities caused by alcohol exposure in FASD.

## RESULTS

### Voluntary maternal drinking during pregnancy causes long-lasting changes to DNA methylation in resulting progeny

The first experiment in this report examines genome-wide differences in DNA methylation in the adult brain [postnatal day (PND) 70] in response to FAE by voluntary maternal drinking. It is followed by assessment of its potential biological effects as outlined below.

#### Global methylation changes to promoterome

**Fig. 1.**
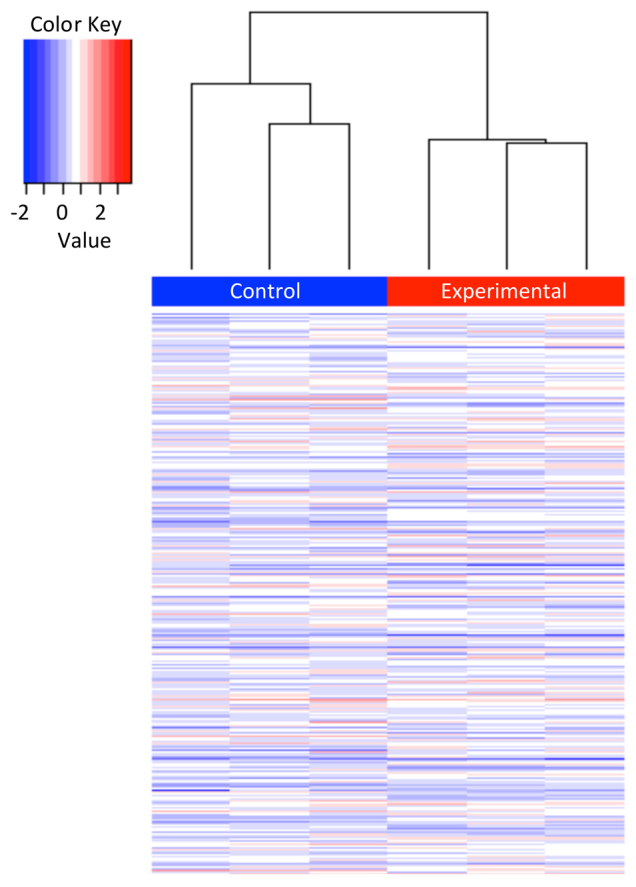
**Hierarchical clustering analysis of differential DNA methylation enrichment peaks from arrays examining the effect of continuous preference drinking on B6 male brains.** To compare differentially enriched regions between ethanol-exposed and control mice, the normalized log_2_-ratio scan values were averaged and then used to calculate the M′ value [M′=Average(log_2_ MeDIPE/InputE) – Average(log_2_ MeDIPC/InputC)] for each probe. NimbleScan sliding-window peak-finding algorithm was run on this data to find the differential enrichment peaks (DEPs).

Results showed that the differences across individuals representing alcohol-exposed and matched control brains, although variable, show a significant (*P*=0.01) effect of FAE on genome-wide DNA methylation. Upon hierarchal clustering (Fig. 1) analysis, it was observed that experimental and control mice group together according to exposure. Furthermore, the results reveal that at least 6660 promoter regions are differentially methylated as a result of FAE.

#### Network analysis

Next, we subjected the identified promoters to Ingenuity Pathway Analysis® (IPA®). This analysis revealed that a large number of genes related to cell death and nervous system development and function are significantly enriched for in fetal-alcohol-exposed brains (Table 1). Network analysis revealed that the ‘Behavior, Neurological Disease, and Psychological Disorders’ network was the most significantly affected network, with an IPA® score of 65. From this network a number of highly connected ‘hub genes’ were identified (supplementary material Fig. S1).

**Table 1.**
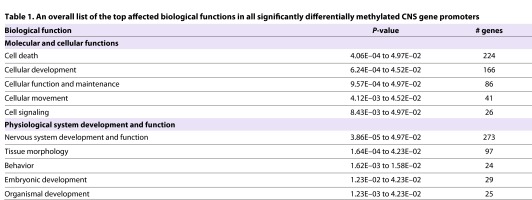
An overall list of the top affected biological functions in all significantly differentially methylated CNS gene promoters Biological function

Among the most prominent hub genes was *App*: its promoter is un-methylated in the treated mice. Furthermore, the promoters of a set of interacting genes (*Akt1*, *Ghr*, *ApoE*, *Ntrk1*) within this hub are also methylated following FAE. Finally, we examined the affected canonical pathways and found that the top two pathways were *Cdk5* signaling (*P*=9.01E–7), with 47/78 molecules affected (supplementary material Fig. S2), and *Pten* signaling (*P*=1.9E–06), with 54/95 molecules affected (supplementary material Fig. S3).

#### Functional consequences

**Fig. 2.**
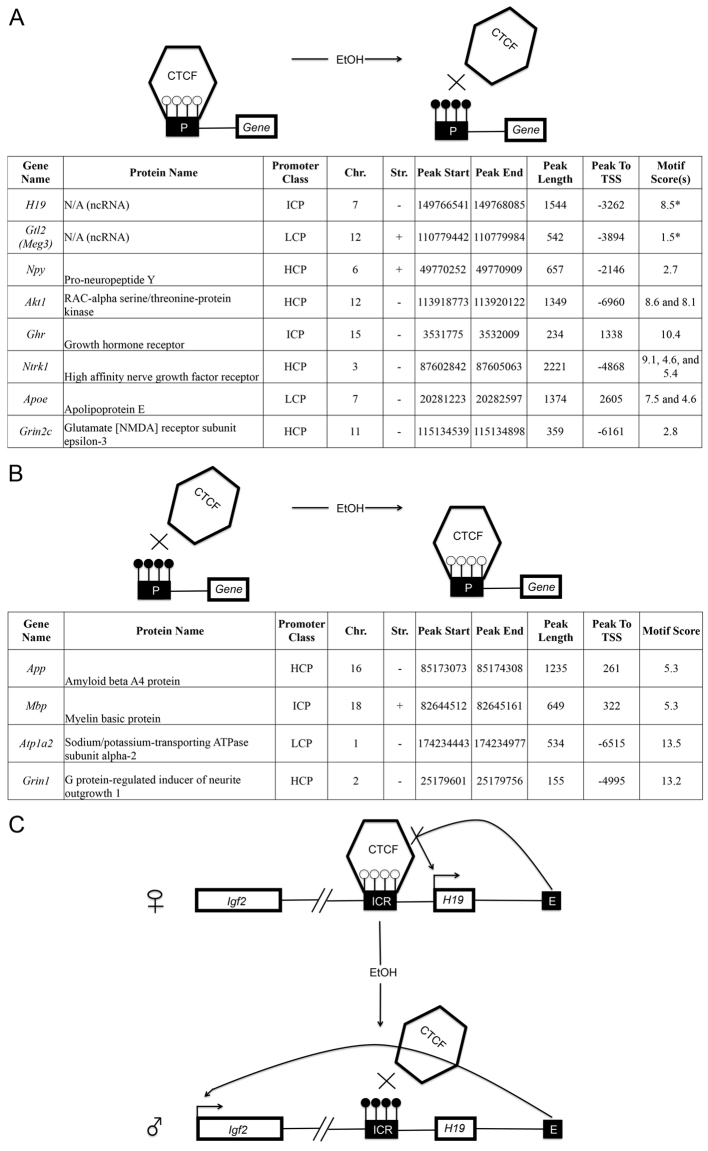
**The functional significance of altered CTCF-binding-site methylation after FAE.** (A,B) Gene promoters (‘P’) with CTCF sites showing increased (A) and decreased (B) methylation after FAE. (C) Schematic of *H19/IGF2* imprinting regulation and the effects of FAE. The black rectangle represents the *H19* ICR, white lollipops represent unmethylated DNA, and black lollipops represent methylated DNA. On the wild-type locus, the ICR exhibits paternal-specific methylation and contains binding sites for CTCF. On the maternal allele, CTCF binds to the ICR and blocks the *Igf2* promoter from accessing the 3′ shared enhancers (E). On the paternal allele, the ICR is methylated, and *H19* transcription is repressed. Because CTCF binding is methylation sensitive, the ICR cannot act as an insulator on the paternal allele, allowing *Igf2* expression to be driven from the enhancer. Our results suggest that, in the case of FAE, imprinting is deregulated owing to increased methylation in the CTCF-binding site, which causes the maternal allele to exhibit paternal imprinting marks.

We then sought to examine whether the changes to DNA methylation occurred in any CTCF-binding sites. Indeed, the same region of *H19/Igf2* identified by Haycock et al. also showed significant (*P*<0.01) differential methylation on our arrays (Fig. 2A). In addition to the *H19*/*Igf2* locus, CTCF binds to differentially methylated regions (DMRs) at a number of other imprinted loci. One of these is the secondary DMR of *Gtl2* (*M*eg3), which also showed significant differential methylation in a CTCF-binding site on our arrays (Fig. 2A).

**Fig. 3.**
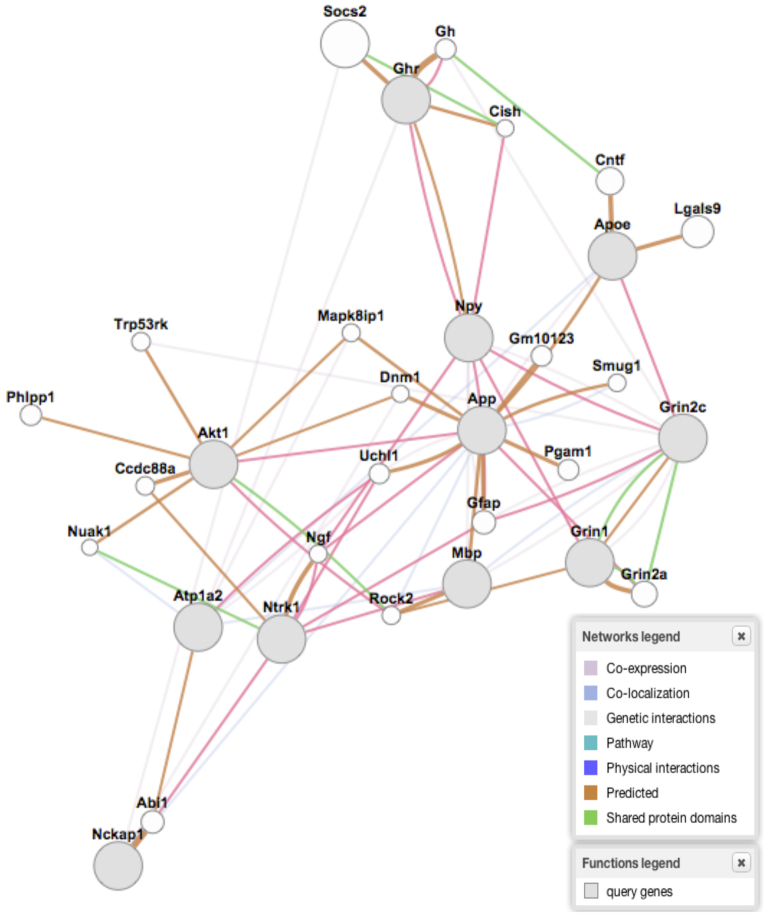
**Gene Mania network analysis of significantly differentially methylated genes containing CTCF-binding sites, from the ‘Behavior, Neurological Disease, and Psychological Disorders’ network.**

Next, we sought to examine whether any of the 30 significantly differentially methylated peaks belonging to different regions of the promoters of major nodes from the ‘Behavior, Neurological Disease, and Psychological Disorders’ network (supplementary material Fig. S1) contained CTCF-binding sites (Bao et al., 2008). Of these 30 regions, 12 (40%) showed sequences that were strongly predicted to be CTCF-binding motifs (Fig. 2A,B). Eight of these promoter regions showed increased methylation and four showed decreased methylation. These genes of interest were then subjected to an independent pathway analysis using GeneMANIA (Warde-Farley et al., 2010), with the results supporting those of IPA® (Fig. 3). However, we note that other methylation-sensitive transcription factors and/or insulators are likely to be involved in the altered transcriptomics resulting from FAE.

### Both voluntary chronic exposure and binge injections at different developmental time points cause long-lasting changes in miRNA and gene expression

The second set of experiments in this report identifies differences in ncRNA and gene expression. We assessed the effect of ethanol via voluntary maternal drinking on the transcripts in the brains of the resulting adult (PND 70) progeny. Furthermore, we also examined binge FAE (injection) at first (T1), second (T2) and third (T3) trimester equivalents of human development in order to confirm and expand on the results.

#### Prenatal ethanol causes changes in the adult brain miRNome

**Fig. 4.**
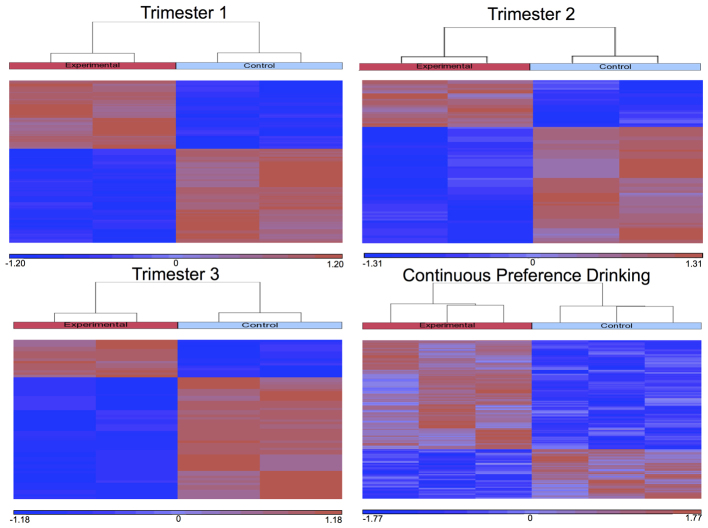
**Heat maps of miRNA expression generated using hierarchical clustering of the four FAE paradigms.** Trimester 1 injection model, trimester 2 injection model, trimester 3 injection model and continuous preference drinking model (ANOVA *P*-value of 0.05 and minimum 1.2-fold cut-off).

The results from mouse miRNA arrays show that FAE resulted in changes to global miRNA expression. The effect of each treatment paradigm was relatively consistent between biological replicates. Consequently, alcohol-treated and matched control expression patterns consistently group together when hierarchically clustered (Fig. 4).

**Fig. 5.**
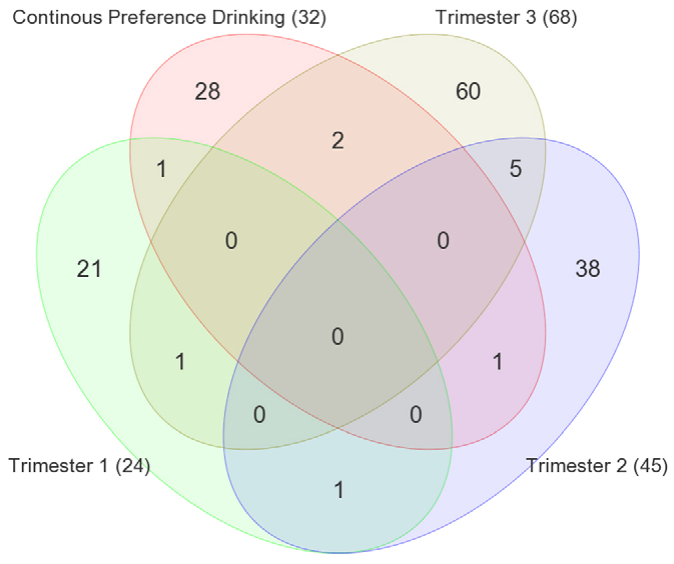
**Venn diagram of common and unique differentially expressed miRNAs identified by four FAE models.** Continuous preference drinking; trimester 1, binge treatment at GD8 and GD11; trimester 2, binge treatment at GD14 and GD16; trimester 3, binge treatment at PD4 and PD7. (ANOVA *P*-value of 0.05 and minimum 1.2-fold cut-off.)

Furthermore, the pattern of expression between alcohol-exposed and matched control brains is quite distinct. We note that the miRNAs that were affected were specific to the developmental timing of alcohol treatment (Fig. 5). Here, the treatment during T1, T2 or T3 resulted in the unique expression for 21/24 (88%), 38/45 (84%) and 60/68 (88%) of the affected miRNAs for each of the three trimesters, respectively. This number for the voluntary consumption [continuous preference drinking (CPD)] paradigm was also comparable [28/32 (88%)].

#### miRNA and target gene alterations upon FAE via voluntary maternal consumption

Next, we used Ingenuity’s miRNA Target Filter® to analyze all possible miRNA and target-gene interactions from the miRNA and gene expression array data sets for the voluntary maternal consumption paradigm. The results were filtered based on the confidence of interaction, brain specificity, and an inverse miRNA to target mRNA relationship (supplementary material Table S1). Overall, 34 genes from the gene expression arrays showed inverse pairwise relationships with 1–13 miRNAs from the miRNA expression arrays that were predicted to target them.

Of the 34 identified target genes, four (*Pten*, *Nmnat1*, *Slitrk2* and *Otx2*) are of particular interest in the context of FASD, owing to their roles in the brain (Table 2), as discussed later. Next, we subjected the 34 genes to IPA®. The analysis revealed a number of fundamental biological processes that were significantly affected, including: a role for seven molecules in cellular development, a role for seven molecules in embryonic development and a role for six molecules in developmental disorders (Table 3). The four genes of interest (*Pten*, *Nmnat1*, *Slitrk2* and *Otx2*) are significant players in these pathways, which suggests a newly identified role for these genes in FASD.

**Table 2.**
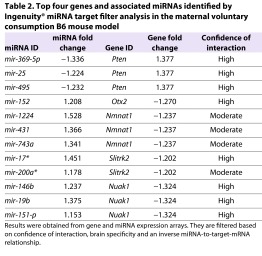
Top four genes and associated miRNAs identified by Ingenuity® miRNA target filter analysis in the maternal voluntary consumption B6 mouse model

**Table 3.**
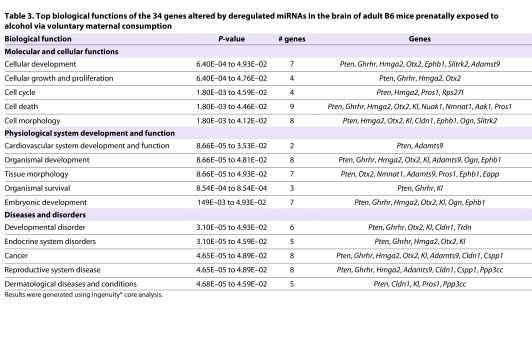
Top biological functions of the 34 genes altered by deregulated miRNAs in the brain of adult B6 mice prenatally exposed to alcohol via voluntary maternal consumption

#### Confirmation experiments

We then sought to confirm the results observed on the two independent array platforms by quantitative PCR (qPCR). Although the results showed similar fold changes to the arrays for all genes of interest examined (*Pten*, *Nmnat1*, *Slitrk2* and *Otx2*), they were not statistically significant (*P*=0.122, *P*=0.129, *P*=0.452 and *P*=0.078, respectively).

However, given the current technological limitations of qPCR (see Discussion), we sought other avenues of confirmation for the miRNA arrays. In subsequent experiments we used an independent platform (Affymetrix mouse gene 1.0 ST expression arrays) for three of the four paradigms, because the gene expression arrays also contained different probes for both mouse-specific snoRNAs and some miRNAs. The results showed that a large number of ncRNAs are similarly affected on the two (miRNA and gene expression) arrays. For example, the *MBII-52* snoRNA, belonging to *Snrpn-Ube3a*, is upregulated, whereas *MBII-85* and snoRNA genes from the *Dlk1-Dio3* region are downregulated.

**Fig. 6.**
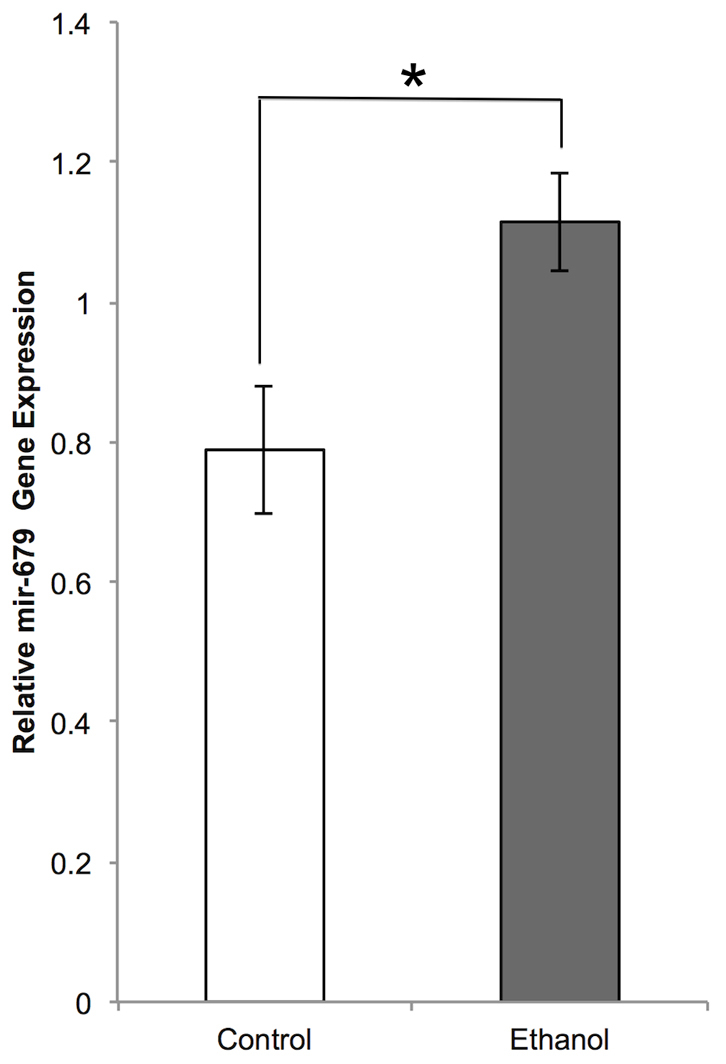
**A bar graph depicting the quantitation of *mmu-mir-679-5p* expression in control and fetal alcohol-exposed (CPD) adult brains.** The *y*-axis depicts the relative mir-679 expression normalized to snoRNA 202, expressed as a mean ± s.e.m. of both biological (*n*=6) and technical (*n*=3) replicates. *P<0.05.

Despite difficulties with confirming gene expression, we were able to validate the results of miRNA arrays of the voluntary consumption model by qPCR. *mir-679-5p*, which is located in the *Dlk1-Dio3* region, showed a 1.45-fold increase (*P*=0.019) in mice that were treated with ethanol during neurodevelopment via maternal voluntary drinking (Fig. 6). The results provide support for the two independent (miRNA and gene expression) array platforms that showed 1.21 (*P*=0.03)- and 1.41 (*P*=0.04)-fold increases, respectively. Ultimately, these results suggest that the long-term changes in ncRNA expression following FAE are subtle and treatment specific, with the exception of *MBII-52*, and must be interpreted with caution given current technological limitations.

### Bioinformatic investigation of the interactions between epigenome and genome

The third set of experiments in this report examines the correlation between all three array-based assays using bioinformatic tools in order to generate a number of novel hypotheses.

#### Alterations in DNA methylation partially correlate to altered expression in the voluntary consumption model

First, we assessed the observed differential methylation of the promoters in relation to the altered expression of protein-coding transcripts (129) and miRNAs (33) in alcohol-exposed brains and their matched controls. A total of 16% (21/129) of the transcripts and 18% (6/33) of the miRNAs that showed significant (*P*<0.05) differential expression (fold change >±1.2) on the expression arrays also showed significant (*P*=0.01) differential methylation in their promoters (supplementary material Table S2). Furthermore, 50 of ∼100 known imprinted genes in the mouse genome and 28 imprinted miRNAs from the *Dlk1-Dio3* region of chromosome 12 showed changes in methylation following FAE (supplementary material Table S3).

#### Genomic mapping of altered miRNA genes in all models

Finally, we mapped the altered miRNAs from the four exposure paradigms to chromosomal regions on the mouse genome. We also assessed them in relation to the mouse imprinting catalog (http://www.mousebook.org/catalog.php?catalog=imprinting). Interestingly, 8/32 (25%) of identified miRNA transcripts in the voluntary consumption treatment paradigm, 2/24 (8%) in the T1 paradigm, 13/45 (29%) in the T2 paradigm and 13/68 (19%) in the T3 paradigm mapped to three known imprinted regions of the mouse genome. The genomic locations include the *Sfmbt2* region of mouse chromosome 2, the *Snrpn-Ube3a* region of chromosome 7 and the *Dik1-Dio3* region of chromosome 12.

## DISCUSSION

By their very nature the results included in this report offer advantages as well as limitations. Among the limitations are the resolution of molecular observations that are subtle and the complexity of our animal model. Indeed, a lack of statistically significant qPCR results casts reasonable doubts on the expression array results and suggests that some of the results are false positives and that there are a number of variables that must also be considered. First, we note that, although qPCR is the gold standard of confirming gene expression changes, the current technology contains a vital limitation related to experiments of this nature. Specifically, it is not sensitive enough to accurately detect low, but statistically significant, fold changes (<2.0) (Peirson and Butler, 2007; Vikalo et al., 2010) that are typical of the fine-tuning nature of miRNAs (Moazed, 2009). Second, we note that most results followed the same trend, up or down, similar to the array results. Finally, the aforementioned results might also represent the heterogeneous nature of whole brain tissue (Ernst et al., 2007; Soverchia et al., 2005; Sun et al., 2012) and the timing and dosage of FAE. These factors create the potential for a large amount of biological variation within the treatment groups that is not considered in the statistics. Thus, instead of relying on qPCR, our interpretation takes a different approach and is based on our observations from multiple exposure paradigms and multiple independent array technologies. Consequently, we leave the interpretation of these results to the reader by discussing both the strengths and weaknesses of the data sets presented.

However, we note that our results follow those of Wang et al., who also observed an alteration to *mir-10b* in fetal brains exposed to alcohol (Wang et al., 2009). Indeed, the pathways and genes identified in this report do not represent a random sample. Rather, they are significantly enriched for FASD-related endophenotypes. Finally, we also acknowledge that it is not easy to establish a one-to-one relationship between a miRNA and a gene because hundreds of miRNAs can regulate a single gene and a single miRNA can regulate hundreds of genes (Bartel, 2009; Mukherji et al., 2011); thus, our current understanding of the relationship between miRNAs and genes is still in its infancy.

With respect to experimental complexity and interpretation, it is worth noting that the results are on an animal model and not on humans. Also, there are limitations in matching the exact dose of alcohol in the two types of alcohol treatments, maternal preference drinking during pregnancy and injection of pups. Given that the mothers in the voluntary consumption paradigm had continuous (24 hour) access to ethanol and water, we did not assess blood alcohol concentration (BAC), owing to variations in consumption patterns between females and to avoid additional stress to pregnant dams related to blood-taking procedures (Kleiber et al., 2012). Although the precise BACs reached were unknown, pregnant females in this study consumed comparable volumes of alcohol (results not shown) to other continuous consumption models that produced peak blood alcohol concentrations of ∼80–120 mg/dl per day (Middaugh et al., 2003). Furthermore, other maternal consumption models with varying degrees of alcohol access have shown that pregnant B6 females will typically consume enough alcohol to reach pharmacologically significant BACs (Allan et al., 2003; Boehm et al., 2008; Caldwell et al., 2008), representing continuous moderate exposure with daily punctuated peaks high enough to result in significant neurocellular damage (Young and Olney, 2006b). This is also supported by the subtle but consistent behavioral changes we have observed in FAE offspring in our previous studies.

Finally, the data analysis is based on the effects of alcohol exposure at different stages of neurodevelopment rather than acting as an assessment of any dose response. Regardless of the potential for differences in blood alcohol levels achieved within and between the paradigms, the results obtained using these mouse models serve as an effective means to examine the long-term consequences of FAE to the developing brain at distinct time points (Clancy et al., 2001; Rice and Barone, 2000). Indeed, despite the aforementioned limitations, this research still advances the understanding of FASD because previous research into FASD has focused on cell lines, which are not appropriate for epigenetic experimentation, specifically in the case of genomic imprinting (Velker et al., 2012), and prior *in vivo* studies into the epigenetics of FASD have focused on whole embryo tissue (Liu et al., 2009). Ultimately, given these facts, we believe that our observations on adult whole-brain tissue are a substantial contribution to the literature and will help to focus future research into specific brain regions using the biomarkers identified.

### Global changes to DNA methylation and related functional consequences

The results included in this report identify that prenatal alcohol causes significant changes in DNA methylation in the developing brain that lasts to adulthood (Fig. 1). It covers a relatively large number (∼6600) of gene promoters that are related to molecular, functional and phenotypic abnormalities implicated in FASD (Table 1). The most significant network identified (score 65) is that of ‘Behavior, Neurological Disease, and Psychological Disorders’, which has a distinct set of ‘hub genes’. Among the most prominent hub genes affected is *App* (amyloid precursor protein), which is a protein that helps direct the migration of neurons during early development (Priller et al., 2006). Interestingly, these results are backed by the literature (Liu et al., 2009) and argue that the crucial gene hubs (*Akt1*, *Ghr*, *ApoE*, *Ntrk1*) identified here (see Results) have the potential to play a crucial role in the manifestation of FASD-related effects. Such results support the relevance of the observed ethanol-induced gene methylation changes in FASD.

The results at hand also allow us to hypothesize on the potential mechanisms that could underlie the biological consequences. We argue that an alteration in promoter methylation might interfere with transcriptional machinery as demonstrated by the role of differential methylation of the *H19* promoter (Hark et al., 2000). Interestingly, we also identified an identical methylation peak in the *H19* promoter (Fig. 2A) that is involved in CTCF binding and imprinting (Lin et al., 2011). This specific peak has previously been identified as showing significant differential methylation in FASD placental tissue (Haycock and Ramsay, 2009). Logistically, CTCF binds to the *H19/Igf2* ICR in a DNA-methylation-sensitive manner and mediates the insulator activity of the unmethylated maternal ICR by blocking the *Igf2* promoter from engaging enhancers downstream of *H19* that are shared by *H19* and *Igf2* (Fig. 2C) (Hark et al., 2000). The deletion or mutation of the four CTCF-binding sites within the ICR causes a paternalization of the maternal allele, *Igf2* biallelic expression and *H19* repression (Engel et al., 2006).

In addition to the *H19* locus, CTCF binds to DMRs at a number of imprinted loci. One of these is the secondary DMR of *Gtl2* (*Meg3*) (Nowak et al., 2011), which also showed differential methylation at a CTCF-binding site. Given the role of CTCF in these two important genes implicated in FASD, we sought to examine whether other important genes that were affected shared this regulatory motif in their promoters. From the ‘Behavior, Neurological Disease, and Psychological Disorders’ network, 30 significantly differentially methylated peaks were examined for CTCF-binding sites using the CTCFBS prediction tool (Bao et al., 2008). Of these 30 regions, 12 (40%) showed sequences that represented predicted CTCF-binding motifs (Fig. 2A,B). Eight of these promoter regions showed increased methylation and four showed decreased methylation. Among the most prominent hub genes was *App*, which is a protein that helps direct the migration of neurons during early development (Priller et al., 2006). Here, its promoter is unmethylated in the treated mice. Furthermore, the promoters of a set of interacting genes (*Akt1*, *Ghr*, *ApoE*, *Ntrk1*) within this hub are also methylated following FAE. These results on *H19* and *App* follow previous research (Liu et al., 2009). However, our research is the first to expand on such genes and show these changes in adult brain tissue long after FAE. The 12 genes identified were then subjected to a pathway analysis (Fig. 3) using GeneMANIA (Warde-Farley et al., 2010), with the results supporting those of IPA (supplementary material Fig. S1).

### Global and specific changes in ncRNA expression

The results of this report also show that FAE affects ncRNA expression in the adult brain (Fig. 4). It is seen in binge injection models as well as the voluntary maternal consumption model [labeled in Figs 4 and 5 as continuous preference drinking (CPD)]. These results suggest that the long-term changes in miRNA expression are dependent on the treatment paradigm (Fig. 5).

The exception to this pattern is the *MBII-52*-specific snoRNA expression, which is affected regardless of the timing of exposure (supplementary material Table S4). Furthermore, in the case of the voluntary maternal consumption paradigm (and possibly the three binge paradigms), FAE is associated with global changes to DNA methylation. These results expand on our hypothesis that a complex residual ‘footprint’ of FAE exists long after exposure (Kleiber et al., 2012).

A large number of studies suggest that the observed alterations in the brain transcriptome (including the miRNome) that follow FAE have a number of possible explanations. First, neuroscience-based brain-imaging studies suggest that they might reflect the result of selective cell death (apoptosis) that changes the proportions of different cell types in the developing brain (Archibald et al., 2001; Autti-Rämö et al., 2002; Gil-Mohapel et al., 2010; Ikonomidou et al., 2000; Klintsova et al., 2007). The elimination of susceptible cell types would result in an altered cellular composition, leading to a distorted transcriptome (which includes the miRNome) (Kleiber et al., 2011). However, although even low blood-alcohol levels can trigger apoptosis in the developing brain (Young and Olney, 2006a), FASD-related behaviors are commonly observed in individuals with no obvious brain abnormalities (Mattson and Riley, 1998; Spadoni et al., 2007), which suggests that a reduction in brain cell populations probably does not account for all the variation in FASD endophenotypes.

A second explanation, put forth by molecular biologists, is that epigenetic modifications could be directly affected by alcohol, given that alcohol is known to affect the biochemical pathway of one-carbon metabolism, which is involved in establishing and maintaining epigenetic marks (Haycock and Ramsay, 2009). Such modifications might include DNA methylation and ncRNAs, which subsequently affect gene expression, and form the focus of this report.

Third, and perhaps most likely, is that the observed changes could result from some combination of the aforementioned two mechanisms. If the changes in DNA methylation and ncRNAs are involved in alteration of gene expression (Kleiber et al., 2012), they must begin early, at the time of FAE. Such changes will be established early, with the marks being passed on through successive cell divisions and accounting for long-term effects. The results included in this report expand on epigenetic studies carried out by molecular biologists in the past decade (Haycock and Ramsay, 2009; Miranda, 2011), while relying on the fundamentals provided by neuroscientists over the past 40 years (Berman and Hannigan, 2000; Mattson et al., 2011).

### Biological consequences of the observed epigenetic changes

Because we examined gene expression, miRNA expression and DNA methylation in a single treatment paradigm [voluntary maternal consumption (CPD)], we were able to examine for any relationships among these three observations. Interestingly, 34 genes, from the gene expression arrays, showed 104 inverse pairwise relationships with 1–13 (31 total) miRNAs, from the miRNA expression arrays, which were predicted to target them (supplementary material Table S1). Of the 34 genes identified by the miRNA target filter, four (*Pten*, *Nmnat1*, *Slitrk2* and *Otx2*) are of special interest in the context of FASD because of their roles in the brain (Table 3).

**Fig. 7.**
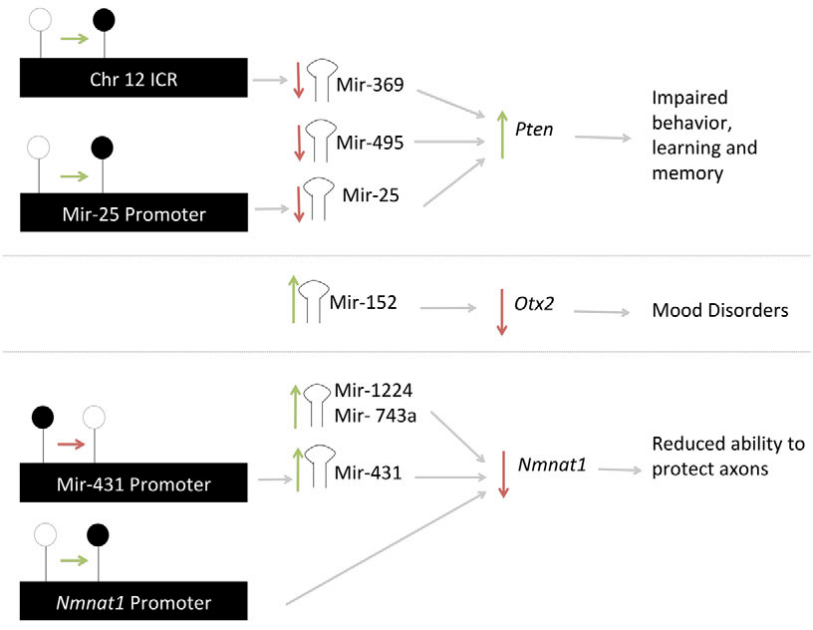
**Summary of select observed epigenetic associations.** White lollipops indicate an absence of DNA cytosine methylation, whereas black lollipops indicate its presence. Green arrows indicate an increase of either methylation or expression, whereas red arrows indicate a decrease.

The first, *Pten*, was upregulated (1.38-fold change and *P*=2.5E–03) and the three miRNAs (*mir-369-5p*, *mir-25* and *mir-495*) predicted to target it were downregulated, with *mir-369-5p* belonging to the *Dlk1-Dio3* cluster (Fig. 7). Additionally, 57% of the molecules involved in the *Pten* signaling pathway showed significant (*P*=1.9E–06) differential methylation (supplementary material Fig. S3). Pten functions as a lipid phosphatase that counteracts the kinase function of phosphatidylinositol-3-kinase (Pi3k) and suppresses *Akt* activation (Maehama and Dixon, 1998). *Akt*, which showed a gain of methylation at a predicted CTCF-binding site (Fig. 2A), is a major mediator of signaling pathways in response to a large spectrum of extracellular stimuli. Upon its activation in neurons, *Akt* phosphorylates different substrates, which in turn regulate diverse processes of neuronal development, including morphogenesis, dendritic development, synapse formation and synaptic plasticity (Yoshimura et al., 2006), all of which are altered in FASD. Studies in mice, with a targeted inactivation of *Pten* in differentiated neurons, showed abnormal social interaction and exaggerated responses to sensory stimuli (Kwon et al., 2006). Given the integral role of *Pten* in neurodevelopment, it is no surprise that it has been implicated in the developmental basis of many major psychiatric disorders (Kim et al., 2009) and now FASD.

The second gene of interest is *Nmnat1*, which was downregulated (–1.24-fold change and *P*=0.01), whereas its three predicted target miRNAs (*mir-1224*, *mir-431* and *mir-743a*) were upregulated (Fig. 7). *Nmnat1* protects against axonal degeneration following mechanical or toxic insults by delaying axonal degeneration (Sasaki et al., 2006). More recent results support its role in protection of key brain structures, such as the hippocampus, that are affected in FASD (Verghese et al., 2011). The third gene of interest (*Slitrk2*) was downregulated (–1.20-fold change and *P*=2.15E–04), whereas two of its predicted miRNAs (mir-17* and mir-200a*) were upregulated. Significant expression of *Slitrk2* is detected only in the adult brain (Aruga and Mikoshiba, 2003). Furthermore, *Slitrk2* is uniquely expressed in immature neurons, and has an inhibitory effect on neurite outgrowth. Finally, *Otx2* was downregulated (–1.27-fold change and *P*=0.02) along with one of its predicted miRNAs (*mir-152*) being upregulated (Fig. 7). *Otx2* is expressed in the brain, is involved in mood disorders (Acampora et al., 1995; Sabunciyan et al., 2007) and was identified in our previous study on long-term brain gene expression changes in FASD (Kleiber et al., 2012).

On the basis of these results, we hypothesize that altered expression in a set of crucial genes with potential to contribute to the FASD endophenotypes (Fig. 7) following FAE involves transcriptional (methylation) as well as post-transcriptional (miRNA) regulation. Furthermore, the similarity and variability in the manifestation of FASD could be attributed to the common (*MBII-52*) and different ncRNAs and genes affected as a result of variability in exposure.

### Genomic loci of interest

We have also observed that ∼20% of the ncRNAs affected are encoded by one of the three imprinted regions of the mouse genome: the *Sfmbt2* (murine 2qA1), *Snrpn-Ube3a* (murine 7qC/human 15q11-q13) and *Dlk1-Dio3* (murine 12qF1/human 14q32.2) regions, which also showed differential methylation in a number of regulatory sites (supplementary material Table S5). We have recently written an in-depth review on these regions (Laufer and Singh, 2012); however, here we will provide a brief description.

The murine *Sfmbt2* region contains developmentally regulated neuron-specific transcripts and has a large cluster of miRNAs (Kagami et al., 2008). *Snrpn-Ube3a* expresses a neuron-specific polycistronic transcript that includes two clusters of snoRNAs, *HB/MBII-52* and *HB/MBII-85* (supplementary material Table S4) (de los Santos et al., 2000; Le Meur et al., 2005; Runte et al., 2001). The *Dlk1-Dio3* locus expresses over 40 miRNAs contained in two clusters, including a cluster of snoRNAs that contains *SNORD113* and *SNORD114* (supplementary material Table S6) (Seitz et al., 2004). The ncRNAs from *Snrpn-Ube3a* and *Dlk1-Dio3* are expressed in embryo, placenta and in the adult, in which their expression is confined mostly to the brain (Seitz et al., 2004). The *Snrpn-Ube3a* and *Dlk1-Dio3* regions have also been associated with several neurodevelopmental disorders (Leung et al., 2009). Furthermore, all three of the ncRNA clusters are transcribed as a single polycistronic unit (Fiore et al., 2009; Le Meur et al., 2005; Wang et al., 2011) with all transcripts showing similar levels of expression, and differ only as a result of unequal stability (Seitz et al., 2004). This suggests that neurodevelopmental alcohol exposure might alter the regulation of not only individual miRNAs or snoRNAs, but entire clusters of co-regulated ncRNAs. More importantly, this process could involve differences in DNA methylation. Although we found differential methylation in these regions, specific experiments will be needed to establish their significance. Our methylation array results follow those of Liu et al., who have reported that FAE causes differential methylation in *Ube3a*, which is located in the *Snrpn-Ube3a* region (Liu et al., 2009).

The *Sfmbt2*, *Snrpn-Ube3a* and *Dik1-Dio3* regions are of special interest to FASD because they are also known to affect a range of endophenotypes seen in FASD. These include impaired growth, craniofacial abnormalities, and a range of cognitive and behavioral deficits (May and Gossage, 2001). Maternal disomy of the *Sfmbt2* region results in fetal and placental growth retardation, whereas paternal disomy was shown to result in normal fetal growth and placental overgrowth (Kuzmin et al., 2008). Also, the *Snrpn-Ube3a* locus is involved in the classic sister imprinting disorders Prader-Willi syndrome (OMIM: 176270) and Angelman syndrome (OMIM: 105830), both of which display developmental delay and deficits in cognitive function (Knoll et al., 1989; Wagstaff et al., 1992). Interestingly, *H/MBII-52* and *H/MBII-85*, which we found to show altered expression as a result of FAE, are believed to be key players in these disorders (Cassidy et al., 2000; Ding et al., 2008; Skryabin et al., 2007). Furthermore, overexpression of *MBII-52*, in a paternal duplication mouse model, shows poor social interaction, behavioral inflexibility, abnormal ultrasonic vocalizations, and anxiety (Nakatani et al., 2009). Ultimately, given our observance of upregulated *MBII-52* in all four paradigms examined, the confirmation of results across two independent array technologies, and the strikingly similar endophenotypes associated with alterations to it, we believe that *MBII-52* serves as an ideal candidate for future research into FASD.

Our results also show that the *Snrpn-Ube3a* region is not the only brain-specific imprinted region affected by alcohol exposure during development. Indeed, altering the dosage of the imprinted genes at the *Dlk1-Dio3* region has also been shown to cause a range of endophenotypes, from growth deficiencies and developmental defects in the embryo and placenta, to defects in adult metabolism and brain function (da Rocha et al., 2008). It has also recently been shown in a rat model that FAE alters the expression of *Dio3* in the hippocampus, as well as altering related behaviors and physiology (Dietz et al., 2012; Sittig et al., 2011). In the case of our research, *mir-369-5p*, which is predicted to target *Pten* (both of which were deregulated in this study), also belongs to this imprinted cluster. Given the role of *Pten* in the brain, it comes as no surprise that alterations to the miRNAs in this cluster could have such profound effects in the brain (Fig. 7).

In conclusion, we report selective alterations in DNA methylation and miRNA expression in adults that were exposed to alcohol during neurodevelopment. The results show that the genes affected as a result of neurodevelopmental alcohol exposure are long lasting and do not represent a random sample. They represent genes involved in genomic imprinting and for which alterations of gene expression have been implicated in endophenotypes related to FASDs. Such observations, being reported for the first time in adult brain following FAE, add support to a role for epigenetic alterations in FASD, while also identifying a number of important biomarkers that warrant future research.

## MATERIALS AND METHODS

### Mice

Male and female C57BL/6J (B6) mice were originally obtained from Jackson Laboratories (Bar Harbor, ME) and maintained at the Health Sciences Animal Care Facility at the University of Western Ontario (London, Ontario, Canada). The Animal Use Subcommittee of the University of Western Ontario approved all procedures undertaken on the animals. B6 mice were housed in standard cages at 21–24°C with 40–60% humidity at a 14-hour light/10-hour dark cycle with access to food and water *ad libitum*. Virgin females of ∼8 weeks of age were time-mated and assessed for pregnancy using the presence of vaginal plugs [gestational day (GD) 0]. From conception (GD0) until weaning at postnatal day (PD) 21, the pups were housed in individual cages with their mothers.

### Alcohol treatment

#### Alcohol treatment by injections (trimester 1, trimester 2 and trimester 3)

Pregnant dams were subcutaneously injected with two 2.5 g/kg body weight doses of ethanol in 0.15 M saline (alcohol-treated) spaced 2 hours apart (at 0 hours and 2 hours), or with saline alone (control), at GD8 and GD11 (trimester 1) or GD14 and GD16 (trimester 2) (Ikonomidou et al., 2000). Control and alcohol-treated dams were age- and weight-matched. Given that the third trimester human equivalent occurs postnatally in mice (Dobbing and Sands, 1979), a binge exposure during this neurodevelopmental period was modeled by treating pups directly on PD4 and PD7 via subcutaneous injection (trimester 3). In this model, pups from one litter were matched across treatment groups for sex and weight to control for litter effects. Injections represented two doses of 2.5 g/kg body weight spaced 2 hours apart with matched controls receiving 0.15 M saline. All resulting offspring were weaned on PD21 and housed in cages with two to four same-sex littermates.

#### Alcohol exposure by CPD

Pregnant females were placed in individual cages, given free access to 10% ethanol and water for 2 weeks to establish a stable drinking pattern, then time-mated and provided both ethanol and water from GD0 to PD10 as described previously (Kleiber et al., 2011). Control dams had access to water only. Voluntary maternal alcohol consumption was measured daily from GD0 to PD10, and females drinking less than 2 ml of alcohol per day were excluded from the study. Resulting pups, both alcohol-exposed and matched controls, were weaned at PD21 and housed in same-sex colony cages of two to four mice.

### Tissue collections and nucleic acid isolation

Alcohol-treated and matched control adult males (PD70) resulting from the four treatment paradigms (*n*=12 per paradigm with six alcohol-exposed and six matched controls) were sacrificed using CO_2_ and cervical dislocation. Whole brains were extracted, snap frozen in liquid nitrogen, and stored at −80°C until RNA and DNA isolation. Whole-brain total RNA was isolated from frozen tissues using TRIzol® Reagent (Invitrogen, Carlsbad, CA), purified using the RNeasy® Mini Kit (QIAGEN, Valencia, CA), and quantified using a NanoDrop ND-1000 spectrophotometer (Thermo Fisher Scientific Inc., Wilmington, DE). Total RNA quality was assessed using the Agilent 2001 Bioanalyzer (Agilent Technologies Inc., Palo Alto, CA). Further, whole brain DNA was isolated from the interphase layer of TRIzol using sodium citrate, followed by ethanol precipitation and purification using the QIAamp® DNA Micro Kit (QIAGEN, Valencia, CA). DNA was then quantified using a NanoDrop ND-1000 spectrophotometer (Thermo Fisher Scientific Inc., Wilmington, DE) and all samples had OD_260_/OD_280_ nm ratios of 1.8–2.0 and OD_260_/OD_230_ nm ratios of 2.0–2.4.

### miRNA expression analysis

Equal amounts of total RNA from non-littermate males (three for the injection models and two for the CPD model) were pooled per biological replicate to reduce litter effects, with no litter contributing more than one individual. Two biological replicates (arrays) were used for each injection model, and three for the voluntary maternal drinking (CPD) model. All sample labeling, hybridization and GeneChip processing was performed at the London Regional Genomics Centre (Robarts Research Institute, London, Ontario, Canada). Briefly, 1 μg total RNA from each treatment paradigm was labeled using the Flash Tag Biotin HSR kit (Genisphere, Hatfield, PA) and hybridized to Affymetrix miRNA 2.0 arrays for 16 hours at 45°C. Probe level (.CEL file) data was generated using Affymetrix Command Console v1.1. Probes were summarized to gene level data in Partek Genomics Suite v6.6 (Partek Inc., St Louis, MO) by using the RMA algorithm (Irizarry et al., 2003). Partek software was used to determine differences between control and ethanol-treated samples using one-way ANOVA and corresponding *P*-values and fold changes. For each treatment model, the miRNAs present on this array were filtered using stringency criteria of 1.2-fold change (*P*=0.05). The selected miRNAs were subjected to a hierarchical clustering analysis by using Euclidean distance and average linkage to assess consistency in ethanol response between the arrays of different treatment paradigms. Furthermore, the chromosomal locations for all miRNAs were determined by using miRBase and Ensembl (Flicek et al., 2011; Kozomara and Griffiths-Jones, 2011). The miRNA expression array results for all treatment protocols were deposited within the NCBI Gene Expression Omnibus (GEO) database under accession GSE34413.

### Expression array hybridization

Single-stranded complementary DNA (sscDNA) was prepared from 200 ng of total RNA as per the Ambion WT Expression Kit for Affymetrix GeneChip Whole Transcript WT Expression Arrays (Applied Biosystems, Carlsbad, CA) and the Affymetrix GeneChip WT Terminal Labeling kit and Hybridization User Manual (Affymetrix, Santa Clara, CA). Total RNA was first converted to complementary DNA (cDNA) and followed by *in vitro* transcription to make cRNA. Subsequently, 5.5 μg of sscDNA was synthesized, end-labeled and hybridized for 16 hours at 45°C to Mouse Gene 1.0 ST arrays (Affymetrix, Santa Clara, CA). GeneChip Fluidics Station 450 performed all liquid handling steps, and GeneChips were scanned with the GeneChip Scanner 3000 7G using the Command Console v1.1 (Affymetrix, Santa Clara, CA). All hybridizations were performed by the London Regional Genomics Center at the Robarts Research Institute, University of Western Ontario. Probe level (.CEL file) data were generated using the Affymetrix Command Console v1.1. Probes were summarized to gene level data in Partek Genomics Suite v6.5 (Partek Inc., St Louis, MO) by quantile normalization using the RMA algorithm adjusted for GC content (Irizarry et al., 2003) and log_2_-transformed. Partek software was used to determine gene level ANOVA *P*-values and fold changes using a Chi-square test. All arrays were analyzed by using a 1.2-fold cut-off with a significance threshold of *P*=0.05. The gene expression array results for the trimester 1, trimester 3 and CPD protocols were deposited within the NCBI GEO database under accessions GSE34469, GSE34549 and GSE34305, respectively (trimester 2 gene expression data was not available).

### Quantitative PCR validation of *mir-679-5p*

cDNA was reverse transcribed from 1 μg of RNA from the voluntary maternal consumption paradigm (*n*=6) and matched controls (*n*=6) using the Applied Biosystems TaqMan™ MicroRNA Reverse Transcription Kit (Foster City, CA) and sequence-specific stem-loop reverse transcription primers from TaqMan™ MicroRNA Assays (Foster City, CA) according to the manufacturer’s protocol. All qPCR primers and miRNA-specific TaqMan™ probes were selected by using the Applied Biosystems (Carlsbad, CA) search engine to identify previously characterized TaqMan™ MicroRNA Assays. snoRNA 202 was chosen as an endogenous control (Gao et al., 2010). The target and control reactions were run in separate tubes on the same plate for each sample as per the manufacturer’s protocol. Three technical replicates were averaged for both the endogenous control and gene of interest for each sample. qPCR reactions were performed on the Applied Biosystems StepOne™ Real-Time PCR System 2.0 according to the manufacturer’s protocol. Fold change was calculated using the ΔΔCt method (Schmittgen and Livak, 2008) and statistically analyzed using Applied Biosystems DataAssist™ Software v3.0. Statistical significance was assessed by an unpaired Student’s *t*-test.

### miRNA target filter and pathway analysis

Data from the voluntary maternal consumption paradigm was analyzed through the use of Ingenuity® microRNA target filter [Ingenuity® Systems (www.ingenuity.com)] to generate lists of interactions between genes and miRNAs of interest. Results were filtered based on a moderate or high confidence of interaction (Lewis et al., 2005; Vergoulis et al., 2012), brain specificity, and an inverse miRNA to target mRNA expression relationship. The identified genes were then subjected to Ingenuity® Pathway Analysis.

### Assessing DNA methylation by MeDIP-chip analysis

Equal amounts of brain DNA from two non-littermate males from the voluntary maternal consumption paradigm were pooled per biological replicate (*n*=*3*) to reduce litter effects. All methylated DNA immunoprecipitation (MeDIP), sample labeling, hybridization and processing was performed at the Arraystar Inc. (Rockville, MD). Briefly, genomic DNA was sonicated to 200- to 1000-bp fragments followed by immunoprecipitation of methylated DNA using Biomag™ magnetic beads coupled to mouse monoclonal antibody against 5-methylcytidine. The immunoprecipitated DNA was eluted and purified by phenol chloroform and ethanol precipitation. The total input and immunoprecipitated DNA were labeled with Cy3- and Cy5-labeled random 9-mers, respectively, and hybridized to NimbleGen MM9 DNA Meth 2.1M Deluxe array (Roche NimbleGen Inc., Madison, WI). This array is a single array design that contains CpG islands (MM9) annotated by the UCSC Genome Bioinformatics database (http://genome.ucsc.edu) and RefSeq promoter regions (from about −8.2 kb to +3 kb relative to the transcription start sites) with a coverage of ∼2,100,000 probes. Scanning was performed with the Axon GenePix 4000B microarray scanner.

Raw data on three arrays was extracted as pair files by NimbleScan software (Roche NimbleGen Inc.). Median-centering quantile normalization and linear smoothing by Bioconductor packages Ringo, limma and MEDME was performed. From the normalized log_2_-ratio data, a sliding-window peak-finding algorithm provided by NimbleScan v2.5 (Roche NimbleGen Inc.) was applied to find the enriched peaks with specified parameters (sliding window width: 750 bp; mini probes per peak: 2; *P*-value minimum cut-off: 2; maximum spacing between nearby probes within peak: 500 bp). The identified peaks were mapped to genomic features: transcripts and CpG Islands. The MA plots and box-plots were applied to assess the quality of raw data and effect of normalization. A correlation matrix was used to describe correlation among replicate experiments.

To compare differentially enriched regions between ethanol-exposed (E) and matched control (C) mice, the log_2_-ratio values were averaged and then used to calculate the M′ value [M′=Average(log_2_ MeDIPE/InputE) – Average(log_2_ MeDIPC/InputC)] for each probe. NimbleScan sliding-window peak-finding algorithm was run on this data to find the differential enrichment peaks (DEPs). The differential enrichment peaks, called by the NimbleScan algorithm, were filtered according to the following criteria: (i) at least one of the two groups had the median value of log_2_ MeDIP/Input ≥0.3 and a median value of M′ >0 within each peak region; (ii) at least half of the probes in a peak had the median value of coefficient of variability (CV) ≤0.8 in both groups within each peak region.

Using an R script program, a hierarchical clustering analysis was completed. The probe data matrix was obtained by using PeakScores from DMRs selected by DEP analysis. ‘PeakScore’ is a measure calculated from the *P*-values of the probes within the peak and reflects the significance of the enrichment. This analysis used a ‘PeakScore’ ≥2 to define the DEPs by using the NimbleScan sliding-window peak-finding algorithm. The peak score is a –log_10_ transformed *P*-value, which is the average of the *P*-values for all probes within the peak. Therefore, a ‘PeakScore’ ≥2 means the average *P*-value was ≤0.01.

All the annotated peaks from the result of DEP analysis were extracted and combined for peak filtering. The filtering criteria are set as ‘peaks within BOTH top 20% PeakScore AND top 20% PeakDMValue’. A heat map was plotted based on the log_2_ ratio of probes within the filtered peak regions of each sample and was generated by TMeV4.6.

### Promoterome, pathway and CTCF analysis

The top gene promoters identified from the DNA methylation arrays were then subjected to Ingenuity Pathway Analysis. Given the role of the top affected genes, we sought to examine the global trend. The genes corresponding to all previously identified significantly affected promoters were subjected to an ingenuity core analysis. The Ingenuity Knowledge Base (genes only) was used to examine for direct and indirect relationships. Results were filtered to consider only molecules and/or relationships specific to species (mouse) and tissues (nervous system). From the identified ‘Behavior, Neurological Disease, and Psychological Disorders’ network, 30 significantly differentially methylated peaks belonging to different regions of the promoters of major nodes were examined for CTCF-binding sites using the CTCFBS prediction tool (Bao et al., 2008). The genes identified as having CTCF-binding sites were then subjected to a pathway analysis using GeneMANIA (Warde-Farley et al., 2010).

## Supplementary Material

Supplementary Material
