# Supplementary material for: Long-lasting alterations to DNA methylation and ncRNAs could underlie the effects of fetal alcohol exposure in mice
Source: Dis Model Mech. 2013 Apr 10;6(4):977–92. doi: 10.1242/dmm.010975 (PMC3701217; doi:10.1242/dmm.010975)
Supplement: Supplementary Material [file supp_6_4_977__index.html]

Long-lasting alterations to DNA methylation and ncRNAs could underlie the effects of fetal alcohol exposure in mice — Long-lasting alterations to DNA methylation and ncRNAs could underlie the effects of fetal alcohol exposure in mice — Supplementary Material 

# Long-lasting alterations to DNA methylation and ncRNAs could underlie the effects of fetal alcohol exposure in mice

## DMM010975 Supplementary Material

**Files in this Data Supplement:**

- **Supplementary Material PDF**
