# Supplementary material for: Long-lasting alterations to DNA methylation and ncRNAs could underlie the effects of fetal alcohol exposure in mice
Source: Dis Model Mech. 2013 Apr 10;6(4):977–92. doi: 10.1242/dmm.010975 (PMC3701217; doi:10.1242/dmm.010975)
Supplement: Supplementary Material [file supp_010975_DMM010975.pdf]

Network 1 : Genepromoternames nervous system and hmr 3925 : Genepromoternames filtered to nervous system : Genepromoternames nervous system and hmr 3925

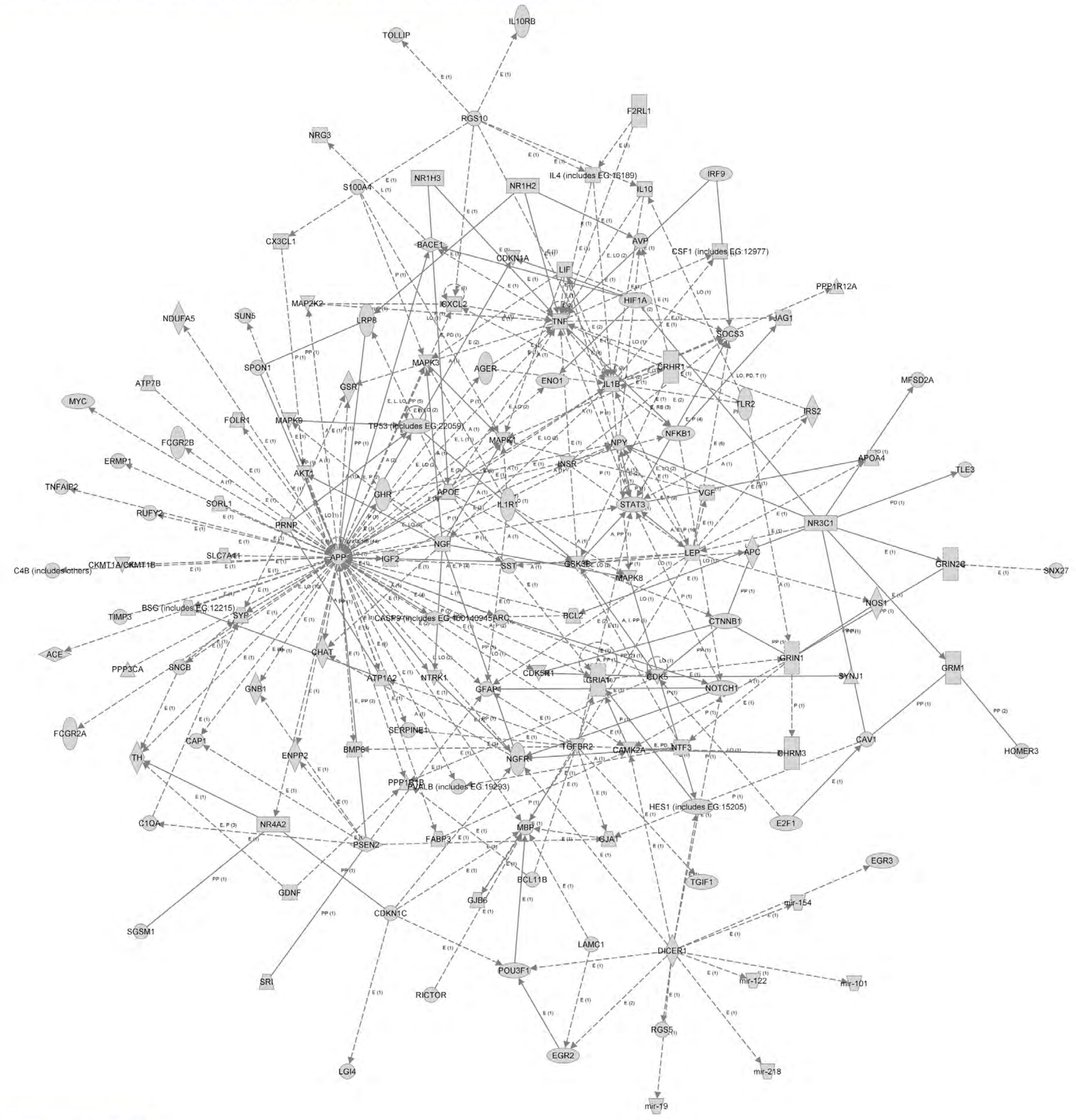

© 2000-2012 Ingenuity Systems, Inc. All rights reserved.

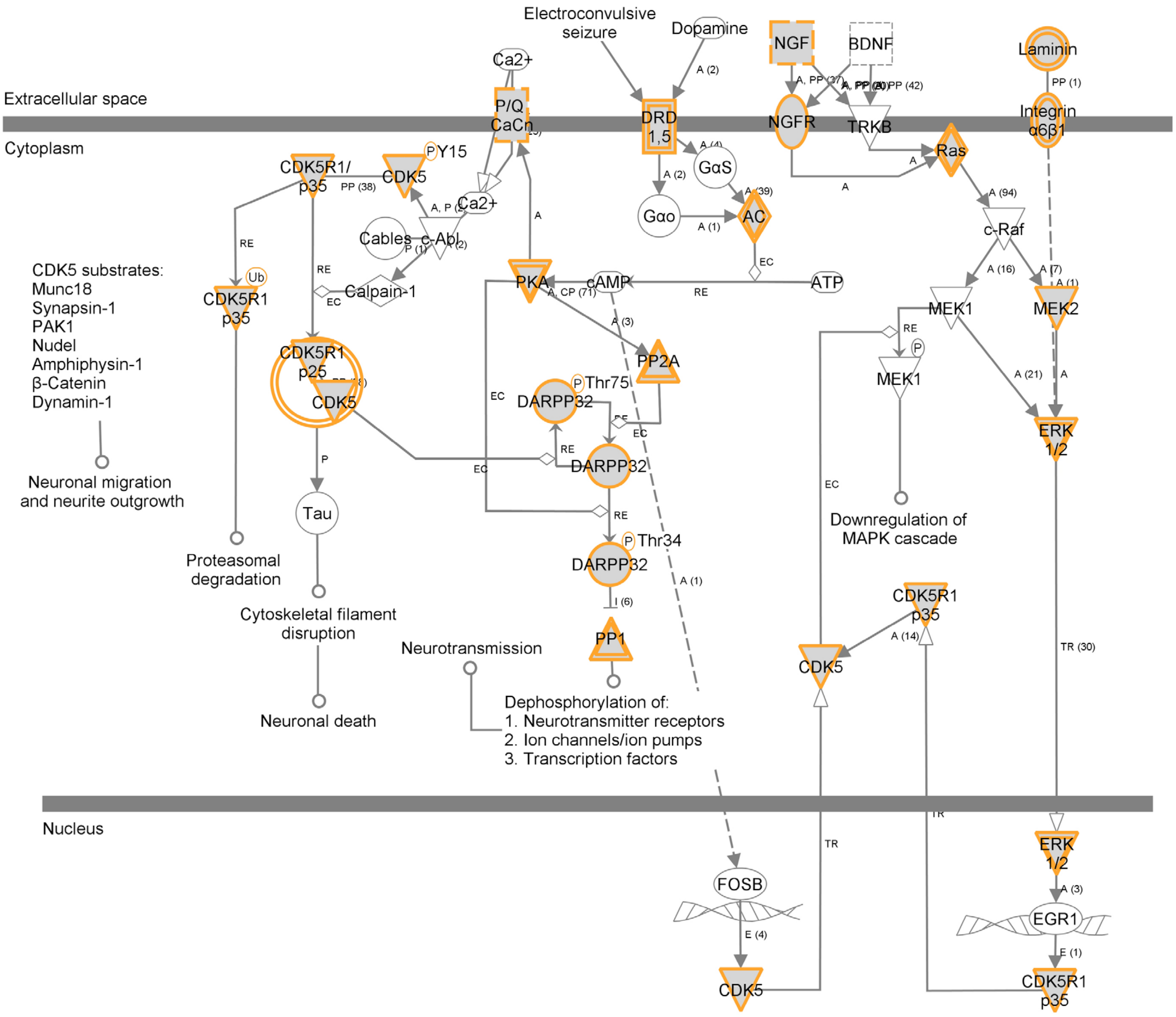

© 2000-2012 Ingenuity Systems, Inc. All rights reserved.

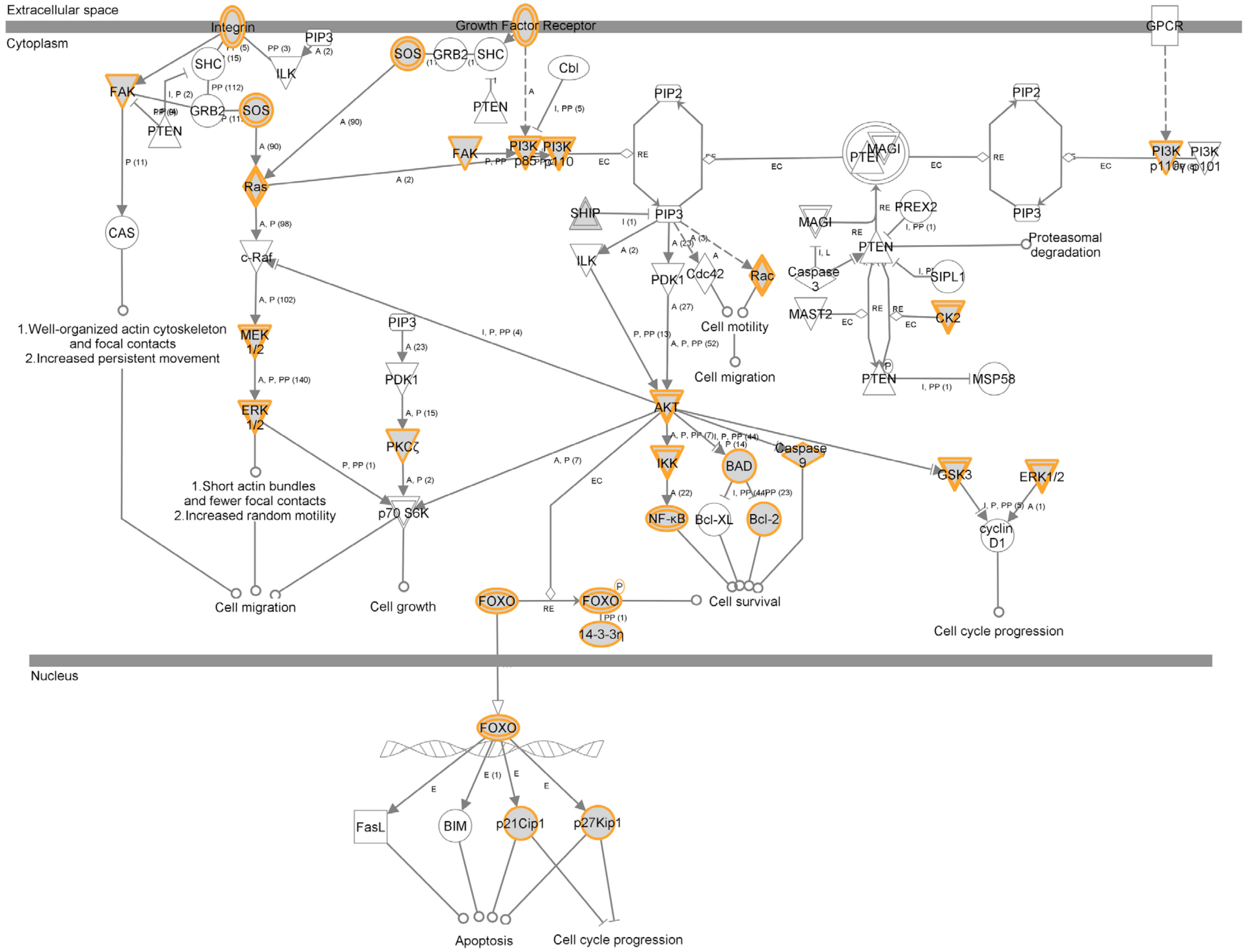

© 2000-2012 Ingenuity Systems, Inc. All rights reserved.

Fig. S1. Top affected network from all significantly differentially methylated promoters identified by IPA; "Behaviour, Neurological Disease, and Psychological Disorders".

Fig. S2. Ingenuity pathway analysis of the *CDK5* signaling pathway. Genes showing significant differential methylation upon FAE are highlighted.

Fig. S3. Ingenuity pathway analysis of the *PTEN* signaling pathway. Genes showing significant differential methylation upon FAE are highlighted.

Table S1. Complete Ingenuity® miRNA target filter analysis of the gene expression and miRNA expression data from maternal voluntary consumption model. Results are filtered based on a moderate or high confidence of interaction, brain specificity, and an inverse miRNA to target mRNA relationship.

miRNA	Fold Change	Gene	Fold Change	Confidence
*let-7d**	-1.463	*Aak1*	1.244	High
*mir-124**	-1.316	*Aak1*	1.244	High
mir-133b	-1.352	*Aak1*	1.244	High
mir-369-5p	-1.336	*Aak1*	1.244	High
*mir-199b**	-1.206	*Aak1*	1.244	Moderate
mir-25	-1.224	*Aak1*	1.244	High
mir-27a	-1.379	*Aak1*	1.244	High
*mir30a**	-1.279	*Aak1*	1.244	Moderate
mir-495	-1.232	*Aak1*	1.244	High
mir-331-3p	-1.174	*Aak1*	1.244	High
mir-34b-3p	-1.334	*Aak1*	1.244	High
mir-362-5p	-1.167	*Aak1*	1.244	Moderate
mir-500	-1.284	*Aak1*	1.244	High
*mir-124**	-1.316	*Adamts9*	1.225	High
mir-369-5p	-1.336	*Adamts9*	1.225	High
mir-25	-1.224	*Adamts9*	1.225	High
*mir-30a**	-1.279	*Adamts9*	1.225	High
mir-495	-1.232	*Adamts9*	1.225	High
mir-34b-3p	-1.334	*Adamts9*	1.225	High
mir-362-5p	-1.167	*Adamts9*	1.225	High
mir-500	-1.284	*Adamts9*	1.225	Moderate
mir-1224	1.528	*Ankrd49*	-1.208	Moderate

mir-489	1.190	*Ankrd49*	-1.208	High
mir-743a	1.341	*Ankrd49*	-1.208	Moderate
mir-452	1.193	*Cdh18*	-1.245	Moderate
mir-151-5p	1.153	*Cldn1*	-1.293	Moderate
mir-743a	1.341	*Cldn1*	-1.293	Moderate
*mir-124**	-1.316	*Cspp1*	1.344	High
mir-369-5p	-1.336	*Cspp1*	1.344	High
*mir-199b**	-1.206	*Cspp1*	1.344	Moderate
mir-495	-1.232	*Cspp1*	1.344	High
mir-340-5p	1.217	*Ddit4l*	-1.260	High
mir-743a	1.341	*Ddit4l*	-1.260	Moderate
*mir-200a**	1.178	*Ddit4l*	-1.260	High
mir-27a	-1.379	*Dnajc13*	1.269	High
*mir-30a**	-1.279	*Dnajc13*	1.269	High
*mir-200a**	1.178	*Eapp*	-1.215	High
mir-369-5p	-1.336	*Ephb1*	1.256	High
mir-495	-1.232	*Ephb1*	1.256	High
mir-369-5p	1.336	*Fam155a*	1.329	High
*mir-30a**	-1.279	*Fam155a*	1.329	High
mir-495	-1.232	*Fam155a*	1.329	Moderate
*let-7d**	-1.463	*Ghrhr*	1.242	Moderate
*mir-124**	-1.316	*Gpatch8*	1.243	High
*mir-15a**	-1.184	*Gpatch8*	1.243	High
mir-369-5p	-1.336	*Gpatch8*	1.243	High
*mir-199b**	-1.206	*Gpatch8*	1.243	High
mir-25	-1.224	*Gpatch8*	1.243	High
mir-495	-1.232	*Gpatch8*	1.243	Moderate
mir-331-3p	-1.174	*Gpatch8*	1.243	Moderate
mir-34b-3p	-1.334	*Gpatch8*	1.243	Moderate
mir-423-5p	-1.159	*Gpatch8*	1.243	Moderate
*let-7d**	-1.463	*Hmga2*	1.220	Experimentally Proven
*mir-15a**	-1.184	*Hmga2*	1.220	High
mir-369-5p	-1.336	*Hmga2*	1.220	High
mir-25	-1.224	*Hmga2*	1.220	High
mir-495	-1.232	*Hmga2*	1.220	Moderate
mir-362-5p	-1.167	*Hmga2*	1.220	Moderate
mir-19b	1.375	*Kl*	-1.271	Moderate
mir-151-5p	1.153	*Kl*	-1.271	Moderate
mir-669h-3p	1.608	*Kl*	-1.271	Moderate
mir-504	1.258	*Kl*	-1.271	Moderate
mir-743a	1.341	*Kl*	-1.271	Moderate

*mir200a**	1.178	*Kl*	-1.271	Moderate
mir-369-5p	-1.336	*Mmrn2*	1.214	Moderate
mir-1224	1.528	*Nmnat1*	-1.237	Moderate
mir-431	1.366	*Nmnat1*	-1.237	Moderate
mir-743a	1.341	*Nmnat1*	-1.237	Moderate
mir-146b	1.237	*Nuak1*	-1.324	High
mir-19b	1.375	*Nuak1*	-1.324	High
mir-151-5p	1.153	*Nuak1*	-1.324	High
mir-146b	1.237	*Ogn*	-1.236	Moderate
mir-669h-3p	1.608	*Ogn*	-1.236	Moderate
*mir-200a**	1.178	*Ogn*	-1.236	Moderate
mir-152	1.208	*Otx2*	-1.270	High
mir-369-5p	-1.366	*Polr3e*	1.204	Moderate
mir-340-5p	1.217	*Ppp3cc*	-1.212	Moderate
mir-1224	1.528	*Pros1*	-1.239	Moderate
mir-743a	1.341	*Pros1*	-1.239	Moderate
mir-369-5p	-1.336	*Pten*	1.377	High
mir-25	-1.224	*Pten*	1.377	High
mir-495	-1.232	*Pten*	1.377	High
mir-19b	1.375	*Rps27l*	-1.206	Moderate
mir-743a	1.341	*Rps27l*	-1.206	High
*mir-15a**	-1.184	*Slc24a1*	1.206	High
mir-27a	-1.379	*Slc24a1*	1.206	High
*mir-17**	1.451	*Slitrk2*	-1.202	High
*mir200a**	1.178	*Slitrk2*	-1.202	Moderate
*mir-17**	1.451	*Tbc1d17*	-1.269	High
mir-146b	1.237	*Tmem19*	-1.231	High
mir-340-5p	1.217	*Tmem19*	-1.231	High
mir-369-5p	-1.336	*Tmem217*	1.200	Moderate
mir-743a	1.341	*Tmem60*	-1.219	Moderate
mir-151-5p	1.153	*Tmem98*	-1.225	Moderate
mir-133b	-1.352	*Trdn*	1.208	Moderate
mir-369-5p	-1.336	*Trdn*	1.208	High
mir-27a	-1.379	*Trdn*	1.208	Moderate
*mir-30a**	-1.279	*Trdn*	1.208	High
mir-495	-1.232	*Trdn*	1.208	High
mir-331-3p	-1.174	*Trdn*	1.208	Moderate
mir-369-5p	-1.336	*Ypel1*	1.222	High
*mir-199b**	-1.206	*Ypel1*	1.222	High
mir-27a	-1.379	*Ypel1*	1.222	High
*mir-30a**	-1.279	*Ypel1*	1.222	Moderate

Table S2. Genes and miRNAs (identified by respective arrays) that showed significant change in both DNA methylation and expression in the voluntary consumption (CPD) paradigm of FAE in B6 mice.

Accession	Gene Name	Methylation in Control	Methylation in Experimental	Change in Expression	Chromosome
NM_001004363	Nuak1	1	1	-1.32	10
NM_001025244	Krtap31-2		1	1.22	11
NM_001033464	Efcab4b		1	1.26	6
NM_001166595	Eif5a	1		-1.20	11
NM_001166590	Eif5a	1		-1.20	11
NM_001166596	Eif5a	1		-1.20	11
NM_181582	Eif5a	1		-1.20	11
NM_001166594	Eif5a	1		-1.20	11
NM_001166593	Eif5a	1		-1.20	11
NM_008788	Pcolce	1		-1.24	5
NM_010441	Hmga2	1	1	1.22	10
NM_011758	Zfp39		1	-1.26	11
NM_173016	Vat1l		1	-1.22	8
NM_023275	Rhoj		2	-1.21	12
NM_023331	Mrpl46	1		-1.36	7
NM_025456	Eapp		1	-1.22	12
NM_025475	Haus2	1		-1.24	2
NM_025718	Dnase1l2		1	1.26	17
NM_053127	Pcdhb2		1	-1.23	18
NM_133435	Nmnat1	1		-1.24	4
NM_133964	Dohh	2	1	-1.25	10
NM_144813	Slc24a1		2	1.21	9
NM_153081	Slc16a11	1		-1.21	11
NM_001110160	Lrrc61		1	-1.20	6
NM_197992	Pcgf1	1		-1.26	6
NM_206867	Gm13280		1	1.24	4
NR_035503	Mir1982	1		1.42	10
NR_029811	Mir199b	1		-1.21	2
NR_029815	Mir19b-1		1	1.37	14
NR_030577	Mir574		1	1.28	5

NR_030492	Mir711		2	1.28	9
NR_030428	Mir762	1	1	2.22	7

Information on mature mRNAs expression was taken from gene expression arrays and information on miRNA was obtained from miRNA expression arrays (1.2 Fold change and p=0.05). Data on significant (p=0.01) methylation differences was obtained from DNA methylation arrays. DNA methylation data is presented as unique peaks seen in either control or experimental samples

Table S3. Imprinted Genes and miRNAs Showing Differential DNA Methylation in the Voluntary Maternal Consumption Paradigm in B6 mice.

Accession	Gene Name	Methylation in Control	Methylation in Experimental	Chromosome
NM_146250	Gpr1		1	1
NM_028673	Zdbf2	1		1
NR_003258	Gnas	1	1	2
NM_001077510	Gnas	1	1	2
NM_201616	Gnas	1	1	2
NM_019690	Gnas		2	2
NM_201618	Gnas	1	2	2
NM_025543	Mcts2	1	1	2
NR_002846	Nespas		2	2
NM_180960	Nnat		1	2
NM_198854	Dlx5		1	6
NM_001135093	Klf14		1	6
NM_008590	Mest		2	6
NM_183308	Pon2	1	1	6
NM_001130191	Sgce		1	6
NR_023846	Apeg3		1	7
NM_007491	Art5		1	7
NM_009876	Cdkn1c	1	1	7
NR_001592	H19		3	7
NM_001122737	Igf2	2		7
NM_001122736	Igf2	1	1	7
NM_010514	Igf2	2		7
NR_002855	Igf2as	2	1	7
NM_008434	Kcnq1		2	7
NR_001461	Kcnq1ot1		2	7
NM_009377	Th		1	7
NM_024290	Tnfrsf23		1	7
NM_138631	Tssc4	1		7
NM_009074	Mst1r		1	9

NM_009538	Plagl1		2	10
NM_016672	Ddc		1	11
NM_010345	Grb10	1		11
NM_010052	Dlk1	1		12
NR_033813	Dlk1	1		12
NR_027651	Meg3		1	12
NR_029542	Mir127		1	12
NR_029550	Mir136		1	12
NR_029564	Mir154		1	12
NR_035440	Mir1906-1		1	12
NR_037313	Mir1906-2		1	12
NR_029590	Mir203		1	12
NR_037232	Mir3071		1	12
NR_037233	Mir3072		1	12
NR_029765	Mir337		1	12
NR_029773	Mir345	1		12
NR_030272	Mir369		1	12
NR_029878	Mir377		1	12
NR_029913	Mir409		1	12
NR_029914	Mir410		1	12
NR_029917	Mir412		1	12
NR_029951	Mir431		1	12
NR_035526	Mir432		1	12
NR_029952	Mir433		1	12
NR_029953	Mir434		1	12
NR_030573	Mir493		1	12
NR_030437	Mir496		1	12
NR_030260	Mir540		1	12
NR_030263	Mir541		1	12
NR_030425	Mir665		1	12
NR_030438	Mir673	1		12
NR_030427	Mir770	2	2	12
NM_184109	Rtl1		1	12
NM_010076	Drd1a	1		13
NM_172812	Htr2a		1	14
NR_002864	Peg13		1	15
NM_027052	Slc38a4		1	15
NM_001164643	Trappc9		1	15
NM_029640	Trappc9		1	15
NR_027772	Airn	1		17
NM_027455	Qpct	1		17

NM_013667	Slc22a2	1		17
NM_145952	Tbc1d12	1		19
NR_001463	Xist		1	X
NM_001081643	Xlr3b		1	X
NM_021365	Xlr4b		1	X
NM_183094	Xlr4c		1	X

DNA methylation data is presented as unique peaks seen in either control or experimental samples.

Table S4. Dysregulated genes snoRNAs in the *Snrpn-Ube3a* region (chromosome 7) identified by miRNA and gene expression arrays.

	CPD			Trimester 1			Trimester 2			Trimester 3		
miRNA array				ID	Fold Change	p-value	ID	Fold Change	p-value	ID	Fold Change	p-value
	No Significant ncRNAs			HBII-52-48	1.21	0.049	HBII-52-2	1.22	0.044	HBII-52-15	1.45	0.001
				HBII-85-7	-1.32	0.037	HBII-85-22	-1.30	0.001	HBII-85-29	1.42	0.026
				HBII-85-23	-1.76	0.050	HBII-85-26	-1.20	0.039			
				HBII-85-24	-1.36	0.039						
				HBII-85-28	-1.43	0.009						
Expression Array	Ensembl ID	Fold Change	p-value	Ensembl ID	Fold Change	p-value				Ensembl ID	Fold Change	p-value
MBII-52	101805	1.4	0.019	97244	1.39	0.009	*Arrays Not Run*			102015	1.46	0.003
	101980	1.22	0.003	101805	1.22	0.050				102017	1.31	0.017
	101860	1.32	0.035	101861	1.22	0.050				102028	1.65	0.001
	101803	1.22	0.002	101896	1.33	0.004				102037	1.42	0.025
	97244	1.28	3.16E-04	101917	1.32	0.017				102046	1.27	0.022
	101917	1.23	0.038	101935	1.32	0.017						
	101951	1.29	0.017	101935	1.49	0.029						
	101896	1.22	2.09E-04	101980	1.28	0.028						
	101932	1.22	2.06E-04	102037	1.33	0.038						
	101816	1.43	0.004									

The Ensembl IDs were shortened by removing the ENSMUST0000000 from the beginning of them.

Table S5. Dysregulated imprinted miRNAs encoded in the *Sfmbt2* region (chromosome 2) identified by miRNA expression arrays.

CPD			Trimester 1			Trimester 2			Trimester 3		
ID	Fold Change	p-value	ID	Fold Change	p-value	ID	Fold Change	p-value	ID	Fold Change	p-value
miR-466f-3p	1.37	0.024	hp_mmu-mir-669j	1.72	0.002	miR-466b-3p	-1.35	0.038	miR-467b	1.25	0.038
miR-467c	-1.23	0.01				miR-669a	-1.78	0.05	miR-467a-1*	-1.31	0.037
miR-467a*	1.87	0.007				miR-466e-3p	-1.49	0	hp_mmu-mir-297c	-1.27	0.037
miR-466c-3p	1.23	0.003				miR-466c-3p	-1.9	0.03	hp_mmu-mir-466e	-1.71	0.034
miR-669h-3p	1.61	0.005				hp_mmu-mir-297b	-1.44	0.031	hp_mmu-mir-466f-2	-1.6	0.046
hp_mmu-mir-466h	1.28	0.027				hp_mmu-mir-297c	-1.56	0.016	hp_mmu-mir-466h	-1.58	0.03
						hp_mmu-mir-466	-1.31	0.028	hp_mmu-mir-669j	-1.44	0.001
						hp_mmu-mir-466f-2	-1.26	0.045	hp_mmu-mir-669m-1	-1.42	0.019
						hp_mmu-mir-669d	-2.11	0.027			

Hairpin precursors contain “hp_” before the miRNA

Table S6. Dysregulated ncRNAs from the *Dlk1-Dio3* region (chromosome 12) identified by miRNA and gene expression arrays.

	CPD			Trimester 1			Trimester 2			Trimester 3		
miRNA array	ID	Fold Change	p-value	ID	Fold Change	p-value	ID	Fold Change	p-value	ID	Fold Change	p-value
	miR-679	1.21	0.03	miR-665	1.16	0.047	miR-666-5p	-1.19	0.003	miR-665	-1.21	0.032
	miR-300	-1.32	0.048				miR-369-5p	1.29	0.003	miR-434-3p	1.15	0.007
							hp_mmu-mir-300[C]	1.28	0.021	miR-376b	-1.7	0.043
							hp_mmu-mir-379[C]	-1.24	0.031	miR-544	-1.26	0.022
										hp_mmu-mir-485[C]	-1.25	0.04
Expression Array	Ensembl ID	Fold Change	p-value							Ensembl ID	Fold Change	p-value
SNORD 113	082745	1.30	0.019	*No Significant ncRNAs*			*Arrays Not Run*			082553	-1.46	0.012
	082553	1.27	0.022							082611	-1.46	0.012
	082611	1.27	0.022							082786	1.56	0.038
	082786	1.24	0.036							082792	-1.31	0.005

Hairpin precursors contain “hp_” before the miRNA
The Ensembl IDs were shortened by removing the ENSMUST0000000 from the beginning of them.
